# Supplementary material for: Association of eye strain with dry eye and retinal thickness
Source: PLoS One. 2023 Oct 20;18(10):e0293320. doi: 10.1371/journal.pone.0293320 (PMC10588844; doi:10.1371/journal.pone.0293320)
Supplement: S2 Table — (DOCX) [file pone.0293320.s003.docx]

**S2a Table. Univariable and multivariable logistic regression analyses of dry eye-related signs and retinal thickness in relation to the presence of eye strain.**

| **Characteristics^†^** | **Univariable OR (95% CI)** | ***P* value** | **Age, sex-adjusted OR (95% CI)** | ***P* value** |
| --- | --- | --- | --- | --- |
| Sex | 0.63 (0.56-0.71) | < 0.01 | 0.63 (0.56-0.71) | < 0.01 |
| Dry eye-related signs |  |  |  |  |
| Short tear break-up time | 1.88 (1.67–2.10) | < 0.01 | 1.75 (1.56-1.97) | < 0.01 |
| Corneal epitheliopathy | 1.44 (1.27-1.63) | < 0.01 | 1.34 (1.18-1.52) | < 0.01 |
| Low Schirmer test value | 0.94 (0.69-1.26) | 0.68 | 0.96 (0.71-1.30) | 0.83 |
| Retinal thickness |  |  |  |  |
| Macular ganglion cell complex thickness | 1.30 (1.06–1.58) | < 0.01 | 1.20 (0.98-1.48) | 0.07 |
| Peripapillary retinal nerve fiber layer thickness | 1.15 (0.92-1.43) | 0.21 | 1.05 (0.84-1.32) | 0.65 |
| Full retinal thickness of whole macula | 1.10 (0.84-1.44) | 0.48 | 1.14 (0.86-1.50) | 0.33 |

† Abnormal parameters were defined as follows: tear break-up time ≤ 5 s, Schirmer test value ≤ 5 mm, the presence of corneal epitheliopathy (positive corneal staining). Retinal thickness value was binarized by mean of the present cohort; thicker than mean = 1, thinner than mean = 0. Men = 1, women = 0.

**S2bTable. Univariable and multivariable logistic regression analyses of dry eye-related signs and retinal thickness in relation to the presence of blurred vision**

| **Characteristics** | **Univariable OR (95% CI)** | ***P* value** | **Age, sex-adjusted OR (95% CI)** | ***P* value** |
| --- | --- | --- | --- | --- |
| Sex | 0.75 (0.66-0.85) | < 0.01 | 0.73 (0.63-0.83) | < 0.01 |
| Dry eye-related signs |  |  |  |  |
| Short tear break-up time | 1.85 (1.63–2.10) | < 0.01 | 2.09 (1.48-2.96) | < 0.01 |
| Corneal epitheliopathy | 1.24 (1.08-1.43) | < 0.01 | 1.24 (1.07-1.43) | < 0.01 |
| Low Schirmer test value | 0.94 (0.69-1.29) | 0.72 | 0.91 (0.66-1.25) | 0.56 |
| Retinal thickness |  |  |  |  |
| Macular ganglion cell complex thickness | 0.59 (0.48-0.72) | < 0.01 | 0.64 (0.52-0.80) | < 0.01 |
| Peripapillary retinal nerve fiber layer thickness | 1.07 (0.86-1.33) | 0.50 | 1.16 (0.93-1.46) | 0.17 |
| Full retinal thickness of whole macula | 0.85 (0.66-1.11) | 0.25 | 0.90 (0.69-1.17) | 0.44 |

† Abnormal parameters were defined as follows: tear break-up time ≤ 5 s, Schirmer test value ≤ 5 mm, the presence of corneal epitheliopathy (positive corneal staining). Retinal thickness value was binarized by mean of the present cohort; thicker than mean = 1, thinner than mean = 0. Men = 1, women = 0.

**S2cTable Univariable and multivariable logistic regression analyses of dry eye-related signs and retinal thickness in relation to the presence of photophobia**

| **Characteristics** | **Univariable OR (95% CI)** | ***P* value** | **Age, sex-adjusted OR (95% CI)** | ***P* value** |
| --- | --- | --- | --- | --- |
| Sex | 0.63 (0.54-0.74) | < 0.01 | 0.62 (0.54-0.73) | < 0.01 |
| Dry eye-related signs |  |  |  |  |
| Short tear break-up time | 1.77 (1.53-2.04) | < 0.01 | 1.58 (1.36-1.83) | < 0.01 |
| Corneal epitheliopathy | 1.32 (1.13-1.55) | < 0.01 | 1.25 (1.07-1.47) | < 0.01 |
| Low Schirmer test value | 0.93 (0.66-1.31) | 0.69 | 0.92 (0.65-1.30) | 0.65 |
| Retinal thickness |  |  |  |  |
| Macular ganglion cell complex thickness | 0.86 (0.68-1.08) | 0.19 | 0.80 (0.63-1.02) | 0.07 |
| Peripapillary retinal nerve fiber layer thickness | 1.12 (0.87-1.43) | 0.35 | 1.07 (0.83-1.38) | 0.59 |
| Full retinal thickness of whole macula | 0.88 (0.65-1.19) | 0.42 | 0.91 (0.67-1.23) | 0.55 |

† Abnormal parameters were defined as follows: tear break-up time ≤ 5 s, Schirmer test value ≤ 5 mm, the presence of corneal epitheliopathy (positive corneal staining). Retinal thickness value was binarized by mean of the present cohort; thicker than mean = 1, thinner than mean = 0. Men = 1, women = 0.
