# Supplementary material for: Association of eye strain with dry eye and retinal thickness
Source: PLoS One. 2023 Oct 20;18(10):e0293320. doi: 10.1371/journal.pone.0293320 (PMC10588844; doi:10.1371/journal.pone.0293320)
Supplement: S1 File — (DOCX) [file pone.0293320.s004.docx]

(Form 5)

Notice

June 1, 2018

Masahiko Ayaki

President, Tsukuba Central Hospital

Toru Takeshima

Stamp

#180602

Title: Survey on dry eye related eye symptoms and signs

Principle investigator: Masahiko Ayaki

The result(s) of Committee are as follows.

Decision: Approved.

Note: This approval is an extension of prior approval on December 12, 2014, permission number 141201. This is a cohort study. A part of routine standard-of-care. Regular report required.
