# Supplementary material for: Association of eye strain with dry eye and retinal thickness
Source: PLoS One. 2023 Oct 20;18(10):e0293320. doi: 10.1371/journal.pone.0293320 (PMC10588844; doi:10.1371/journal.pone.0293320)
Supplement: S2 File — (DOCX) [file pone.0293320.s005.docx]

November 12, 2018

Notice

Otake Clinic

Masahiko Ayaki

Chairman,

Institutional Review Board and Ethics Committee of Kanagawa Medical Association

Stamp

Title: Survey on dry eye related eye symptoms and signs

Principle investigator: Masahiko Ayaki

The results of Committee on Oct 18, 2018 are as follows.

Approved.

(approved on November 12, 2018, permission number krec2059006)

Institutional Review Board and Ethics Committee of Kanagawa Medical Association (approved on November 12, 2018, permission number krec2059006) Institutional Review Board and Ethics Committee of Kanagawa Medical Association (approved on November 12, 2018, permission number krec2059006) Institutional Review Board and Ethics Committee of Kanagawa Medical Association (approved on November 12, 2018, permission number krec2059006)

**Study design and participants**

This study was a clinic-based, retrospective, cross-sectional study involving healthy subjects attending Tsukuba Central Hospital from January 2017 to July 2021. The Institutional Review Board and Ethics Committee of the Tsukuba Central Hospital approved this study (approved on December 12, 2014, permission number 141201). The Institutional Review Board and Ethics Committee of Kanagawa Medical Association (approved on November 12, 2018, permission number krec2059006) approved this study and participants were recruited from January 2019 to July 2021 at Otake Clinic Moon View Eye Center.
