# Supplementary material for: Association of eye strain with dry eye and retinal thickness
Source: PLoS One. 2023 Oct 20;18(10):e0293320. doi: 10.1371/journal.pone.0293320 (PMC10588844; doi:10.1371/journal.pone.0293320)
Supplement: S3 File — (PDF) [file pone.0293320.s006.pdf]

平成３０年１１月１２日

審査結果通知書

おおたけ眼科つきみ野医院
綾木　雅彦　殿

公益社団法人 神奈川県医師会
倫理審査特別委員会 委員長　㊞

神奈川県医師会倫理審査特別委員会委員長印

審査対象及び課題名：「ドライアイ症状と眼所見の関連調査」

研究責任者名：おおたけ眼科つきみ野医院　綾木　雅彦

さきに新規申請のあった上記課題に係る臨床研究については、平成 30 年 10 月 18 日に審査（迅速審査）を行い、下記のとおり判定したので通知します。

なお、実施計画書等に変更が生じた場合は、倫理審査特別委員会に報告してください。

記

判　　定	（１）承認）　２）修正したうえで承認　３）条件付き承認<br>４）不承認　５）保留　６）停止　７）中止
理由又は勧告	
そ　の　他	

平成30年11月13日

請　求　書

おおたけ眼科つきみ野医院
綾木　雅彦　　　　　　　　様

公益社団法人神奈川県医師会
会　長　　菊　岡　正　和

神奈川県医師会倫理審査特別委員会委員長印

次のとおりご請求いたします。

請求額<br><br>¥ 10,000 −
請求内容<br><br>10/18 倫理審査手数料として
振込先<br><br>神奈川県医師信用組合　本店<br>普通預金　0016818<br>カナガワケンイシカイ　イッパンカイケイ<br>神奈川県医師会　一般会計<br><br>（振込手数料はご負担ください。）

お問い合わせ
公益社団法人神奈川県医師会
事務局　広・情課　松本
横浜市中区富士見町3-1
電　話　　045-241-7000
