# Supplementary figures and images for: Association of eye strain with dry eye and retinal thickness
Source: PLoS One. 2023 Oct 20;18(10):e0293320. doi: 10.1371/journal.pone.0293320 (PMC10588844; doi:10.1371/journal.pone.0293320)

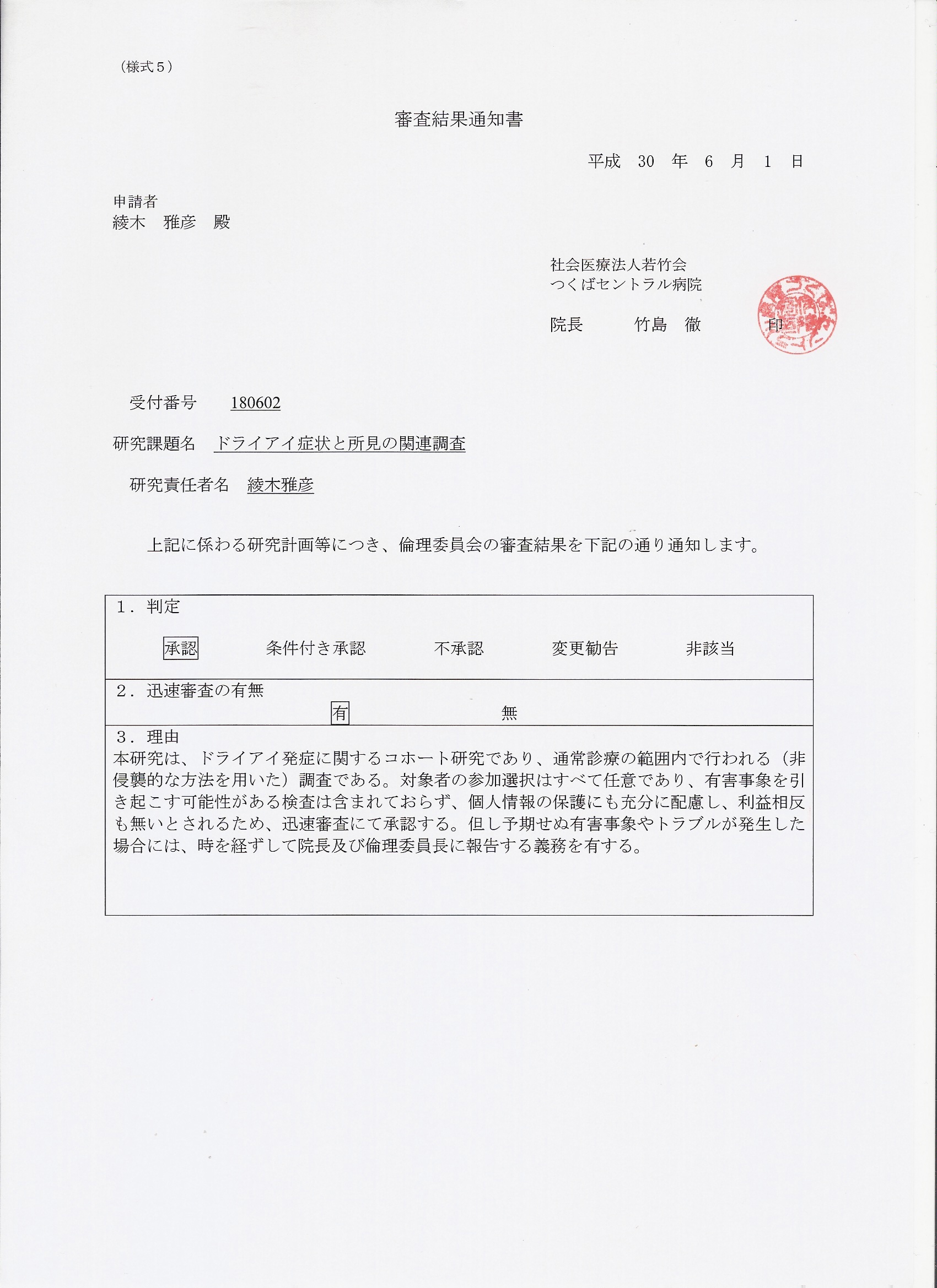

Supplement: S4 File — (JPG) [file pone.0293320.s007.jpg]
