# Supplementary material for: Association of eye strain with dry eye and retinal thickness
Source: PLoS One. 2023 Oct 20;18(10):e0293320. doi: 10.1371/journal.pone.0293320 (PMC10588844; doi:10.1371/journal.pone.0293320)
Supplement: Attachment — Submitted filename: FatigueRes2023-F.docx [file pone.0293320.s008.docx]

Dear Editor and Reviewers

Thank you very much for reviewing our manuscript. To aid in the re-review of this manuscript, we have included a point-by-point response to each comment. The reviewer’s comments are italicized and placed in square brackets. In addition, within the revised manuscript, we have used underlined text to highlight changes in response to the reviewers’ comments.

Masahiko Ayaki, MD

Reviewer’s comments and responses

Reviewer #1

*[Reviewer 1, comment 1] [My comments: Overall, this paper is well-written, demonstrating a significant effort. The study's aims and objectives are deserving of thorough investigation. While the study's foundation appears robust, the lack of clarity in the language employed poses challenges in comprehension. It would be advisable for the authors to engage with a writing coach or copyeditor to enhance the text's coherence and readability.]*

We appreciate the reviewer’s comments. We have amended the text according to the following reviewer’s comments.

 *[Reviewer 1, comment 2] [The study does not adequately connect the existing literature from prior research in this field to its aims and conclusions. It is recommended that the authors revise both the Introduction and Discussion sections to incorporate references to relevant literature, particularly focusing on recently published works.]*

We appreciate the reviewer’s comments. We added recently published works and amended the text as follows.

[Introduction, page 2, line 47]” Eye strain is a common ocular symptom in eye clinic patients with normal best-corrected visual acuity.^1,2^ It is a growing health problem that has been exacerbated during the COVID-19 pandemic due to increased screen time from telework and e-learning.^3-8^ Digital eye strain due to virtual learning and other new technologies is an emerging social burden^4-8^ and the prevalence of eye strain has been reported to be as high as 68.5%^5^ and 81%^6^ among visual terminal display users. Pavel et al.^6^ reviewed and discussed numerous contributing factors for modern eye strain, including environmental and work factors, device-related factors, uncorrected refractive errors, ocular surface disorders, blink, and musculoskeletal modifications. Lotfy et al.^7^ conducted a cross-sectional online survey of 412 participants and multiple logistic regression analysis revealed that studenthood and increased nighttime screen use were independent risk factors for digital eye strain by an odds ratio (OR) of 10.60 and 3.99, respectively. Presbyopia also seems to have developed earlier in life during the COVID-19 pandemic probably due to stress and increased digitalization leading to eye strain even in the younger population.^9^ However, most reports describe review and survey results only and real examination data have not been sufficiently documented. Eye strain is a symptom with multiple presentations, including ocular pain, headache, heaviness, blurred vision, irritation, itching, lacrimation, and photophobia.^5^ It encompasses a broad spectrum of ~~is an umbrella term for~~ vision-related and nonvision-related pathologies, in addition to problems related to visual load, air conditioning, lighting, and ergonomics.^4-8^ ~~Indeed, some patients complain of eye strain of unrecognized origin with no visual task.~~

[Discussion, page 8, line 195] “The present study confirmed the results of recently published works,^4-7^ and linked underlying pathologies with patients’ symptoms by demonstrating ORs calculated with objective data.”

[Discussion, page 10, line 261]” In conclusion, the current study successfully identified female gender, short TBUT, and SPK as significant risk factors for eye strain, blurred vision, and photophobia with considerable ORs, and further confirmed GCC thickening is a risk factor for eye strain. Moreover, the present results contribute to understanding the association of visual symptoms with DE and short-wavelength light activating ipRGC-mediated ocular discomfort and pain and should help in the clinical reasoning and management of common visual symptoms in daily eye practice.”

Added references

5.	AlQarni AM, AlAbdulKader AM, Alghamdi AN, Altayeb J, Jabaan R, Assaf L, et al. Prevalence of Digital Eye Strain Among University Students and Its Association with Virtual Learning During the COVID-19 Pandemic. Clin Ophthalmol. 2023; 17:1755-68.

6.	Pavel IA, Bogdanici CM, Donica VC, Anton N, Savu B, Chiriac CP, et al. Computer Vision Syndrome: An Ophthalmic Pathology of the Modern Era. Medicina (Kaunas). 2023; 59(2):412.

7.	Lotfy NM, Shafik HM, Nassief M. Risk factor assessment of digital eye strain during the COVID-19 pandemic: a cross-sectional survey. Med Hypothesis Discov Innov Ophthalmol. 2022; 11(3):119-28.

8.	Mohan A, Sen P, Shah C, Jain E, Jain S. Prevalence and risk factor assessment of digital eye strain among children using online e-learning during the COVID-19 pandemic: Digital eye strain among kids (DESK study-1). Indian J Ophthalmol. 2021; 69(1):140-4.

9.	Wolffsohn JS, Davies LN, Sheppard AL. New insights in presbyopia: impact of correction strategies. BMJ Open Ophthalmol. 2023; 8:e001122.

*[Reviewer 1, comment 3] [ Abstract: 1. The objectives outlined by the authors in the abstract does consistent with introduction are the titles of the study! I suggest having primary and secondary aims.
Abstract: “The purpose of this study was to investigate the association between the prevalence of abnormal ocular examination results and the common visual symptoms of eye strain, blurred vision and photophobia.”*

*Introduction:” this study aimed to investigate any association between abnormal ocular examination results and the common visual symptoms of eye strain, blurred vision and photophobia, to complement our previous association of eye strain with retinal thickness”]*

We appreciate the reviewer’s comments. We amended the text as follows.

[*Introduction, page 3, line 93*] “In addition, we attempted to identify risk factors for each symptom and explore relevant management for patients with normal vision complaining of visual symptoms”

*[Reviewer 1, comment 4] [Abstract: 2. The conclusion lacks a comprehensive summary of the results.]*

We appreciate the reviewer’s comments. We amended the text as follows.

[Abstract]”Conclusions: The current study successfully identified female gender, short TBUT, and SPK as significant risk factors for eye strain, blurred vision, and photophobia with considerable ORs.”

 *[Reviewer 1, comment 5] [Introduction 1. Lines 50-52 need references.]*

We appreciate the reviewer’s comments. We added references and amended the text in response to comment 2.

*[Reviewer 1, comment 6] [ Introduction 2. Line 49” The authors should use academic language instead of "It is an umbrella term"= it encompasses a broad spectrum of”.*

We appreciate the reviewer’s comments. We have amended the text accordingly.

 *[Reviewer 1, comment 7] [ Methods:1. The demographic data should be incorporated within the Method section.]*

We appreciate the reviewer’s comments. We amended the text as follows.

[Methods, page 4, line 123]” Consequently, a total of 6078 patients (3920 women, mean age 49.0 ± 20.4 years) were eligible for analysis. DE medication was used in 1578 patients (26.0%); hyaluronate in 782 (12.9%), diquafosol in 531 (8.7%), rebamipide in 84 (1.4%), and steroid in 179 (2.9%). ”

*[Reviewer 1, comment 8] [Methods: 2. Additionally, it is advisable to provide more details regarding the yes/no questionnaire, including the total number of questions and their specific content.]*

We appreciate the reviewer’s comments. We amended the text as follows.

[Methods, page 5, line 129]”Participants were interviewed for the presence or absence (yes/no) of three common visual symptoms, namely eye strain, blurred vision and photophobia. These questions were selected from items on the Dry Eye–Related Quality-of-Life Score questionnaire^38^ as the most prevalent symptoms seen in outpatient eye clinics at Keio University Hospital. The questionnaire comprised of three questions: “Do you feel eye strain?”, ”Do you have blurred vision?”, and ”Do you feel sensitivity to light?”.

Added reference

38. Sakane Y, Yamaguchi M, Yokoi N, Uchino M, Dogru M, Oishi T, et al. Development and validation of the Dry Eye-Related Quality-of-Life Score questionnaire. JAMA Ophthalmol. 2013;131(10):1331-8.

*[Reviewer 1, comment 9] [Methods: 3. Furthermore, it would be beneficial to provide references for the statement, "These symptoms were selected as the most prevalent in outpatients visiting the eye clinic of Keio University Hospital in 2012."]*

We appreciate the reviewer’s comments. We have responded to this comment in the answer above.

*[Reviewer 1, comment 10] [ Results: 1. The author might consider utilizing alternative graphical representations to present the data, such as employing bar charts to facilitate a clearer comparison of the characteristics presented in Tables 2, 3, and 4. This approach could enhance the ease of understanding for the readers.]*

We appreciate the reviewer’s comments. We have created graphical representations as follows. Additionally, we transferred Tables 2-4 to supplemental file.

**Figure 1a**

**Figure 1b**

Legend for Figure 1. Univariable logistic regression analyses of dry eye-related signs and retinal thickness in relation to the presence of eye strain, blurred vison, and photophobia. Non-adjusted results are Fig 1a and age and sex adjusted results are Fig1b. **P* < 0.01. Odds ratios and 95% confidence intervals are indicated. Abnormal parameters were defined as follows: tear break-up time ≤ 5 s, Schirmer test value ≤ 5 mm, the presence of corneal epitheliopathy (positive corneal staining). Retinal thickness value was binarized by mean of the present cohort; thicker than mean = 1, thinner than mean = 0. Men = 1, women = 0. Abbreviations: GCC, ganglion cell complex thickness; RNF, retinal nerve fiber layer thickness; Whole macula, full thickness of whole macula.

*[Reviewer 1, comment 11] [ Conclusion: The discussion section should be expanded to create a more comprehensive link between the obtained results and the final conclusion.]*

We appreciate the reviewer’s comments. We have already amended the conclusion text in response to Comment 2.

*Reviewer #2:*
 *[Reviewer 2, comment 1] [This manuscript seeks to determine the risk factors/ association between retinal thickness, ocular surface diseases and measures (SPK, TBUT among others) and common visual symptoms such as eye strain, blurred vision and photophobia. This information is important, as understanding the effects of retinal thickness on the ocular surface examination and symptoms. The manuscript is well written but I have few comments.]*

We appreciate the reviewer’s comments.

 *[Reviewer 2, comment 2] [ Title and abstract: Overall, the title and abstract cover the main aspect of the work. However, the conclusion section of the abstract talks on the risk factors of eye strain only and ignored the risk factors of photophobia and blurred vision.]*

We appreciate the reviewer’s comments. We amended the text as follows.

[Abstract]”Conclusions: The current study successfully identified female gender, short TBUT, and SPK as significant risk factors for eye strain, blurred vision, and photophobia with considerable ORs.”

 *[Reviewer 2, comment 3] [ Introduction: The background and information are relevant to the study.]*

We appreciate the reviewer’s comments.

 *[Reviewer 2, comment 4] [ Methods: Overall, the methods are clear and can be replicated.
Line 100, the sentence does not read well. I would suggest the authors rephrase the sentence.]*

We appreciate the reviewer’s comments. We amended the text as follows.

[Methods, page 4, line 114] “All the data used in this study, including the patient interviews, were collected as part of routine eye care examinations. The authors had access to information that could identify individual participants during and after data collection.”

*[Reviewer 2, comment 5] [ Line 114: The authors provided the year when patients' interviews for common eye symptoms was done (2012); however, the ethical approval was in 2021 for the Keio University and the earliest approval was in 2014. Kindly clarify.]*

We are sorry to have confused you with our ethical approval dates. Patient interviews were conducted from April 2015 to March 2020 in the Tsukuba Central Hospital and from December 2018 to March 2020 in the Otake Eye Clinic. Ethical approval was obtained on 12 December 2014 for Tsukuba Central Hospital and 12 November 2018 for Otake Eye Clinic. The ethical approval granted in 2021 from Keio University was to permit authorship for authors (KN and MA) with appointments at Keio University School of Medicine. We hope this clarifies the situation.

We added a reference and amended the text as follows.

[Methods, page 5, line 129] “Participants were interviewed for the presence or absence (yes/no) of three common visual symptoms, namely eye strain, blurred vision and photophobia. These questions were selected from items on the Dry Eye–Related Quality-of-Life Score questionnaire^38^ as the most prevalent symptoms seen in outpatient eye clinics at Keio University Hospital. The questionnaire comprised of three questions: “Do you feel eye strain?”, “Do you have blurred vision?”, and “Do you feel sensitivity to light?”.

Added reference

38. Sakane Y, Yamaguchi M, Yokoi N, Uchino M, Dogru M, Oishi T, et al. Development and validation of the Dry Eye-Related Quality-of-Life Score questionnaire. JAMA Ophthalmol. 2013;131(10):1331-8.

*[Reviewer 2, comment 6] [ Results; The results are relevant. However, there was no information on the controls mentioned in the results section. I would suggest the authors provide details on the composition of the controls.]*

We appreciate the reviewer’s comments and apologize for the confusion regarding control groups. The comparative analyses were conducted after stratification of the participants by the presence or absence of symptoms and so the “control” groups were the participants without the corresponding symptoms. We have amended the text as follows.

[Results, page 6, line 165] “Regarding retinal thickness, mean GCC was thicker in those with eye strain than in those without eye strain, and thinner in those with blurred vision than in those without blurred vision.”

[Results, page 6, line 170] “Individuals with short TBUT and SPK were more likely to present with eye strain, blurred vision and photophobia, while patients with thick GCC were more likely to present with eye strain, and patients with thin GCC were more likely to present with blurred vision.”

*[Reviewer 2, comment 7] [ Discussion; In the Discussion section, the results are discussed from various angles and placed into context without being overinterpreted. The indicated limitations of the study provide an opportunity to inform future research.]*

We appreciate the reviewer’s comments.

 *[Reviewer 2, comment 8] [ Conclusion; I would suggest a stronger statement at the end.]*

We appreciate the reviewer’s comments. We amended the text as follows.

[Discussion, page 10, line 261]” In conclusion, the current study successfully identified female gender, short TBUT, and SPK as significant risk factors for eye strain, blurred vision, and photophobia with considerable ORs, and further confirmed GCC thickening is a risk factor for eye strain. Moreover, the present results contribute to understanding the association of visual symptoms with DE and short-wavelength light activating ipRGC-mediated ocular discomfort and pain and should help in the clinical reasoning and management of common visual symptoms in daily eye practice.
